# Supplementary material for: Electron–phonon coupling in copper intercalated Bi2Se3
Source: Sci Rep. 2022 Jul 15;12:12097. doi: 10.1038/s41598-022-15909-w (PMC9287361; doi:10.1038/s41598-022-15909-w)
Supplement: Supplementary file 1 — Supplementary Information. [file 41598_2022_15909_MOESM1_ESM.docx]

**Supplementary information**

**Sample structure**

A selected Bi_2_Se_3_ single crystal was contacted with metal electrodes using electron-beam lithography; the structure of the electrodes and the sample are displayed in Fig. S1a, which includes both an optical image and a scanning electron micrograph (SEM). To remove the natural oxide layer before deposition of metal, the TI was subjected to Ar milling procedure at energy 700 eV. This etch removed a few nanometers from the top surface of the Bi_2_Se_3_ crystal as schematically indicated in Fig. S1b. An overdosed PMMA layer was used to protect the side walls of the crystal from metal deposition (dark black rectangular shape in the SEM picture). Aluminium contacts (with 5-nm Ti sticking layer) were deposited on top surface of the single crystal (white/gray colour in the SEM picture) using an ultra-high vacuum e-beam evaporator.


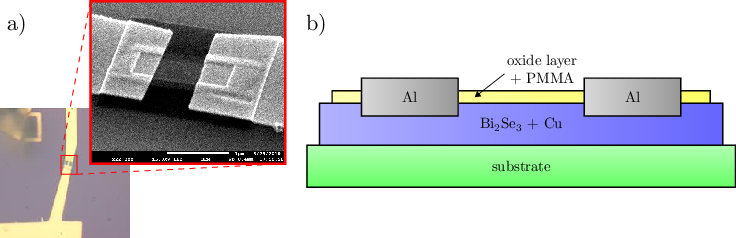


**Fig. S1**. (a) Optical microscope picture of metallic contacts deposited on a Bi_2_Se_3_ sample; the blow-up displays a SEM picture of the metallic contacts on the top surface of the sample. (b) Schematic cross-sectional view of the contacts on the measured device. The etching depth of the Al contacts is a few nanometers; the thickness of the sample amounts to 60 nm. The separation of the 600 x 600 nm^2^ contacts is 700 nm while the Bi_2_Se_3_ sample extends still 300 nm further beyond the contacts (2500 nm in length). The Joule heating is deposited in to the electrons in the Bi_2_Se_3_ crystal between the Al contacts, but strong electronic heat diffusion spreads the heat across the whole crystal.

**Sample characterization using Energy-Dispersive X-ray spectroscopy (EDX)**

Uniform distribution of intercalated copper atoms was confirmed using energy-dispersive X-ray (EDX) spectroscopy. Fig. S2. display a SEM image of the measured sample with the chosen area for the EDX studies. The frames labelled Bi, Se, Cu display the distribution of these three constituents in the sample. The similar distribution of Cu compared with Se and Bi indicates uniform distribution of intercalated copper over the sample.


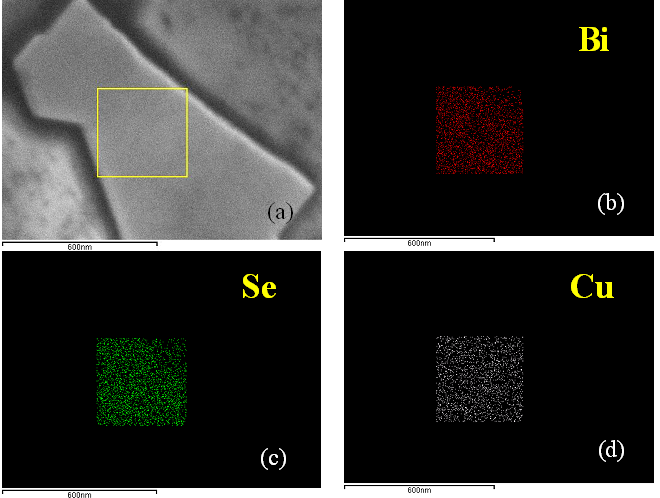


**Fig. S2**. (a) SEM image of a Bi_2_Se_3_ crystal used for EDX measurements: data was collected over the 300 x 300 nm^2^ surface region marked with a yellow square. (b-d) Scan results of EDX spectroscopy showing the distribution of Bi, Se and Cu atoms in the measured, intercalated Bi_2_Se_3_ crystal with line resolution of 1024 x 768.
